# COLLECTING RESIDENT DATA FROM RESIDENTIAL CARE COMMUNITIES USING MAIL QUESTIONNAIRES: A NEW APPROACH

**DOI:** 10.1093/geroni/igz038.2160

**Published:** 2019-11-08

**Authors:** Ozcan Tunalilar, Paula C Carder, Sarah Dys, Sheryl Elliott

**Affiliations:** Portland State University, Portland, Oregon, United States

## Abstract

Researchers who collect resident-level data from RCCs face several challenges. Conducting face-to-face, on-site interviews with administrators is costly and presents scheduling difficulties. Contacting administrators by telephone requires multiple attempts. Even when they are reached, they have limited time and might not share resident-level data. To overcome these difficulties, the current survey of Oregon RCCs combines a mail questionnaire with a self-administered sampling tool that allowed communities to select a random sample of their residents (two from each community). Pilot interviews with administrators indicated they were able to select a random sample of their residents easily, quickly, and accurately using this method. The feasibility and validity of this method were further tested by comparing results to community-level aggregate data collected using traditional mail questionnaires from RCCs (n=392). The similarities and differences between resident- and community-level data are discussed within the context of sampling design and mode of data collection from RCCs.

